# Including Social and Behavioral Determinants in Predictive Models: Trends, Challenges, and Opportunities

**DOI:** 10.2196/18084

**Published:** 2020-09-08

**Authors:** Marissa Tan, Elham Hatef, Delaram Taghipour, Kinjel Vyas, Hadi Kharrazi, Laura Gottlieb, Jonathan Weiner

**Affiliations:** 1 General Preventive Medicine Residency Program Johns Hopkins Bloomberg School of Public Health Baltimore, MD United States; 2 Department of Health Policy and Management Johns Hopkins Bloomberg School of Public Health Center for Population Health Information Technology Baltimore, MD United States; 3 Department of Health Policy and Management Johns Hopkins Bloomberg School of Public Health Baltimore, MD United States; 4 Division of Health Sciences Informatics Johns Hopkins School of Medicine Baltimore, MD United States; 5 Social Interventions Research and Evaluation Network Center for Health & Community University of California San Francisco, CA United States

**Keywords:** social determinants of health, information technology, health care disparities, population health

## Abstract

In an era of accelerated health information technology capability, health care organizations increasingly use digital data to predict outcomes such as emergency department use, hospitalizations, and health care costs. This trend occurs alongside a growing recognition that social and behavioral determinants of health (SBDH) influence health and medical care use. Consequently, health providers and insurers are starting to incorporate new SBDH data sources into a wide range of health care prediction models, although existing models that use SBDH variables have not been shown to improve health care predictions more than models that use exclusively clinical variables. In this viewpoint, we review the rationale behind the push to integrate SBDH data into health care predictive models and explore the technical, strategic, and ethical challenges faced as this process unfolds across the United States. We also offer several recommendations to overcome these challenges to reach the promise of SBDH predictive analytics to improve health and reduce health care disparities.

## Social and Behavioral Determinants of Health and Predictive Analytics

Since the Health Information Technology for Economic and Clinical Health act of 2009, the majority of US health care systems have adopted electronic health records (EHRs) for patient care [1]. Faced with increased financial incentives to improve population health, care coordination, and quality of care, health care providers and payers now use EHRs and other digital data sources to understand how past associations and trends in their patient populations can be used to forecast health care–related outcomes, a component of the widely known strategy of *predictive analytics* [1,2].

Predictive analytics uses extensive data, modeling, and algorithms to predict individual and population events and has a long history in commercial industries [3]. For better or worse, commercial industries have developed innovative techniques to *mine* demographic, socioeconomic, and consumer behavior data as part of the forecasting and analytics process. For example, web-based sellers and banks collect personal information on purchase histories, credit data, consumer behaviors, and life events that are available in various digital databases. These institutions use such data to make predictions for various goals, such as determining ideal customers for specific products or services and how much institutions should offer to whom [4].

There are 2 broad approaches to predictive analytics. The modeling and simulation approach is used to test hypotheses or assess the consequences of scenarios where the rules of the models are developed from theories. Such models also employ data to initialize variables, to calibrate free parameters, or for validation. Alternatively, predictive analytics may also use machine learning in which models are exclusively built from data via algorithms and tested on data that mirror the calibration and validation steps of modeling and simulation, respectively. These approaches can be combined in complex systems [5]. This paper focuses on machine learning and provides several observations that apply to modeling and simulation. Generally, the modeling and simulation approach is useful in systems where the dynamics are well known, whereas machine learning is useful when accurate simulations cannot be performed and there are enough data to determine a model [5]. On the basis of the specific prediction goal, different types of data and methods are required and thus have different associated limitations and challenges.

In health care, the same techniques are used with different goals. Over the last decade, health insurance plans have ramped up the use of predictive analytics, employing patient demographics, insurance claims data, and clinical characteristics derived from EHRs to create statistical models of future health care risks and resource utilization [6]. Analysts have also developed predictive models for health and health care. These data science techniques generally involve larger and more complex databases but represent an application of traditional statistical forecasting methods using a wide range of techniques such as deep neural networks, natural language processing (NLP), random forest, and decision tree algorithms [7,8].

The growing awareness of associations between social and behavioral factors and health has led predictive modeling to explore the incorporation of social and behavioral determinants of health (SBDH) into forecasting [9,10]. For example, on an individual level, diet and physical activity affect health care use and costs [11,12]. At the community level, characteristics of neighborhoods, such as food access and transportation, play significant roles in health outcomes, morbidity, and mortality [13-15].

Although SBDH factors have been incorporated in the predictive modeling process to forecast health care–related outcomes, there are limitations related to the use of such factors. For instance, machine learning methods are not generally developed to capture changing SBDH factors. They mainly address the stationary distributions of the SBDH factors. A change in the data requires providing longitudinal data to the model to perform time series modeling and to capture these changes. If a change in the distribution of data is necessary (eg, to reflect potential trends in SBDH over time), then the approach of modeling and simulation may be used to explore various scenarios. An example is the common use of event-driven simulations in health care research [16].

A growing crop of initiatives uses SBDH to predict health care use in the United States [17]. Although the methods and evidence underlying these new models that incorporate SBDH are nascent and have not shown improved predictions over traditional clinical measures, the medical community’s interest in SBDH needs in conjunction with predictive analytics continues to increase [18,19].

## The Rationale for Including Social and Behavioral Determinants in Predictive Models

Studies in the United States and worldwide have suggested that SBDH, such as educational attainment, have a greater impact on premature mortality than clinical care access and quality [10,20]. A meta-analysis in the United States found that income inequality, social support, segregation, individual and neighborhood poverty, and education level were responsible for 50% of deaths [9]. Some literature on mortality estimates the lack of quality medical care to encompass 10% to 20% of deaths [10,21,22]. Entities such as the World Health Organization have recognized the role of SBDH factors in health equity and committed to action on these determinants [23].

Several national agencies have recognized and advocated for the incorporation of SBDH into health care practices and the standard use of health data. The National Academies of Science, Engineering, and Medicine have identified 5 complementary activities that can facilitate the integration of social care into health care. These activities include the following: “(1) identify the social risks and assets of defined patients and populations; (2) focus on altering clinical care to accommodate identified social barriers; (3) reduce social risk by assisting in connecting patients with relevant social care resources; (4) understand existing social care assets in the community, organize them to facilitate synergies, and invest in and deploy them to positively affect health outcomes; and (5) work with partner social care organizations to promote policies that facilitate the creation and redeployment of assets or resources to address health and social needs.” [24] Moreover, the eHealth initiative, a national coalition focused on health data interoperability in the United States, advocates the use of SBDH data to coordinate care, evaluate interventions that address social needs, identify gaps in community resources, predict health risk, and develop SBDH-sensitive interventions to improve health [25].

### Potential Benefits of Including Social and Behavioral Determinants in Predictive Models

Bolstered by the initiatives of the national organizations, incorporation of SBDH into predictive models could help to (1) identify patients and populations who need more resources, (2) improve health care reimbursement for providers who serve patients with social needs, (3) reduce health and health care disparities, and (4) improve the quality of health care.

Predictive analytics and SBDH risk segmentation could facilitate efforts to identify patients who would benefit from more resources and targeted services. This may lessen the resource burden of universal social risk screening or social care delivery [26]. For example, a systematic risk analysis could help identify patients with modifiable social risks at a higher risk of poor medical outcomes. This type of segmentation could help health systems target appropriate resources, for example, referrals to case management, social service agencies, or government support programs such as the Supplemental Nutrition Assistance Program [27-29]. In addition to using SBDH-sensitive analytics to identify vulnerable individuals, this approach could also help health care organizations or partner agencies identify disadvantaged communities, such as neighborhoods with food deserts [26,30]. A health care system truly desiring to maximize its impact on the health of a community could more effectively increase food access at the neighborhood level by working with farmers’ markets and grocery stores in addition to individual-level interventions.

Under the present federal regulations for Medicaid-managed care, social and behavioral services such as care coordination are reimbursed through capitation. Predictive analytics and SBDH risk segmentation could support new payment models to adequately reflect the medical and social complexity of patients [31]. Beyond capitated or global payments, contextualizing patients with their SBDH needs enables health care payers to more accurately assess providers’ care for vulnerable populations who require more health care resources, thus impacting their fee-for-service payments [27]. Present Medicaid-managed care regulations could support value-added services that would not be reimbursed under capitation alone but would address the health needs of members, such as interventions that assess environmental triggers of asthma [31]. Several states (eg, Rhode Island, Minnesota, and Oregon) have adopted the Accountable Care Organization models that reward health care providers for addressing their Medicaid populations’ SBDH with adjusted payment structures [32,33]. Patient protection laws in the United States regarding insurance denials and premium payments should be upheld to ensure that SBDH risk segmentation does not increase the burden of health care costs to disadvantaged populations [34].

Identifying and accounting for the increased risk of poor health outcomes and associated health care utilization is critical to the elimination of disparities in care for vulnerable populations. The spread of COVID-19 across the United States and worldwide is a great example of how predictive modeling could help health care systems and public health officials address health disparities and potentially change the course of the pandemic. The COVID-19 pandemic has highlighted long-standing health disparities [35,36]; neighborhoods with the highest proportion of racial and ethnic minorities and people living in poverty are experiencing higher rates of hospitalization and death [37-40]. In response, several research teams have started to include information on SBDH in predictive modeling and assessment of COVID-19–related risk and outcomes [39,41].

Exclusion of SBDH-related variables in risk-adjusted reimbursement models would result in lower reimbursement for patients with greater social needs, which dissuades providers from caring for these patients in capitated systems [42]. Employing SBDH in risk-adjusted capitated payment models could translate into improved health care policy by supporting organizations to more effectively meet the needs of individuals and communities with greater social needs.

Beyond payment adjustment, stratifying patients by their SBDH risk levels could reveal health disparities as well as promote health care quality by establishing a mechanism to fairly evaluate providers’ care of patients with social disadvantages [42]. Health systems and payers could further evaluate the quality of health care by developing specific SBDH-dependent quality indicators that bolster equity in health care across the range of patients served [42].

## Present State of Including Social and Behavioral Determinants in Predictive Analytics

Although there is a strong and compelling body of literature on the observed associations between SBDH and health, to date, diagnosis-based forecasting models used to predict cost and utilization have not yet shown the incremental value of adding SBDH risk factors to predictions. Some published reports using community-level SBDH data contribute only slightly to the predictive model performance beyond individual patient characteristics extracted from EHR data [43,44].

Similarly, SBDH-oriented predictive models using newer applications of machine learning techniques have shown varying levels of performance in predictions. A neural network predictive model that incorporates SBDH was found to identify, with 78% accuracy, over two-third of the Medicare patients in their sample who would not respond to automated medication refill requests and may benefit from targeted outreach [45]. Seligman et al [46] applied linear regression and different machine learning techniques to predict systolic blood pressure, BMI, waist circumference, and telomere length using SBDH variables of gender, income, wealth, education, public benefits, family structure, and health behaviors. Although neural networks outperformed other machine learning techniques as fit for their sample, most of their tested machine learning models performed similar to the simpler regression models, and all models had poor out-of-sample prediction [46]. Applying random survival forest methods to develop a predictive model using the poverty status and EHR data, Bhavsar et al [44] did not find that risk prediction for health care services and hospitalization outcomes improved beyond models using traditional EHR data. Similarly, a machine learning model using random forest decision methods on structured and unstructured SBDH only improved sensitivity (67.6%) by 0.1% and showed decreased specificity (69.6%) by 1.9% compared with their tested non-SBDH models in predicting referrals for social needs [47].

Given the evidence-based expectation that SBDH should improve predictive models, why have published predictive models not shown enhanced predictions? Although insufficient data and suboptimal methods are potential explanations common to all research, triple challenges unique to the SBDH context include the diversity of data sources and health outcomes used in existing models as well as the lack of transparency, which together pose an important question about model accuracy.

### Diversity of Data Sources

A wide range of SBDH variables and data sources are used in predictive models and no guidelines exist to distinguish which variables and data sources would best improve the performance of the predictive model. A rapid review of social, behavioral, and environmental determinants of health used with clinical data identified 744 variables among 178 articles, in which the majority of articles included socioeconomic and material conditions [48]. Data sources vary from individual-level EHR data and insurance claims to community-level data from the United States census and similar sources as well as commercial data such as information from credit reporting agencies.

Health plans have historically used insurance claims, which include diagnostic and prior utilization information of varying completeness across health care settings, for predictive modeling to forecast utilization and cost [27,49]. More recently, health payers and other private health care companies have obtained consumer and financial data, such as information on household size, income, and wealth measures, from credit reporting agencies to better assess their members’ needs [27,29,50]. For instance, one company mines public data on education, law enforcement records, birth records, voter registration, and derogatory records such as a history of evictions and liens [27].

Rather than commercial data, academic centers and government organizations have primarily relied on individual-level clinical information derived from structured and unstructured EHRs [51] and relevant risk factors on a community level extracted from public surveys [52], such as the United States Census Bureau American Community Survey, which includes multiple indicators of neighborhood deprivation [43,53]; the Food Access Research Atlas, which describes food deserts [54,55]; and the American Housing Survey, which contains information on housing characteristics [56,57]. In one systematic review of predictive models using EHR data, 36 of the 106 unique studies included SBDH data in one of their final predictive models [58]. However, the social determinants included were limited to race or ethnicity alone in 19 of the 36 EHR-based studies [58]. The same systematic review included behavioral determinants in 30 of these EHR-based predictive models. However, 12 of these studies’ behavioral variables were limited to tobacco use or smoking alone [58]. As another case example, a Kaiser predictive model that uses race and ethnicity as one variable to develop a hypoglycemia risk model omitted race in their final, simpler model on finding that race was not one of the strongest predictors of hypoglycemia compared with clinical factors [59].

In addition to survey-collected data aggregated at the geographic level, academic centers are expanding this community-level framework to include geocentric data such as transit data, which contains data on access to transportation [60], the Environmental Protection Agency’s Air Quality Index data [61], and food desert data from the United States Department of Agriculture’s Food Access Research Atlas [62].

As expected with predictive models, the performance of a model varies depending on the selected SBDH variables and data sources [43,44]. When analyzing SBDH variables, the diversity of data sources has implications for a model’s ability to address challenges associated with SBDH, such as accurately assessing the temporal duration of SBDH and determining the spatial-level effects of population-level SBDH data. Researchers need to critically analyze SBDH variables and data sources to ensure the selection of variables and high-quality data sources that accurately and authentically capture SBDH factors to be tested.

### Diversity of Health Outcomes

Health care–based predictive models that integrate SBDH risk factors have been used to forecast a wide range of health care–relevant endpoints. Although, most often, the predicted outcomes include health care costs and utilization, such as emergency department visits, hospitalizations, and readmissions [27,44,63,64]. There is no consensus on which health outcomes are the most appropriate to predict with specific SBDH factors. Within the public health, academic, and health policy sectors, models have expanded their focus outside the realm of medical care. For example, the Centers for Disease Control and Prevention (CDC), the CDC Foundation, and the Robert Wood Johnson Foundation collaboratively created 500 Cities, a tool that uses community-level socioeconomic characteristics to predict city-level health behaviors, mortality, and morbidity [65,66].

Similar to challenges related to data sources, the diversity of health outcomes as the endpoint for the predictive models will impact assessing the performance of their methods and determining the best methods to address specific SBDH variables or to set the stage for standardized guidelines for specific SBDH variables and outcomes.

### Lack of Transparency

Many predictive models that incorporate SBDH data have been developed and are used in the private sector and are therefore not only proprietary but also unavailable for public review and scrutiny. Consequently, other researchers cannot replicate the methods used in these predictive models. Several predictive modeling companies that have made use of only clinical risk factors now extensively market the inclusion of SBDH data in their predictive risk models [27,29,50]. One company relies exclusively on consumer data, rather than medical data, to develop as many as 70 different models to predict patients at risk for general poor health and high health care costs [67]. For example, one commercial model developer described a case study using its socioeconomic score model to predict the risk of common chronic diseases, highlighting the score’s successful prediction in the top 10% and bottom 10% of the score risk data, although it did not describe how the model performed in the remaining 80% of the population covered [68].

However, the lack of transparency also extends to the academic sector. When data used for a data-driven model, source code, and the model itself are not made open source, the derived models cannot be replicated, a problem known as the *reproducibility crisis* in machine learning [69]. When available, analysts would ideally search out the code and data for models in code repositories to learn how models are organized [70]. However, in a survey of 400 artificial intelligence conference papers with algorithms, only 6% shared the code and about one-third shared their data [69]. Reasons for avoiding sharing range from dependence on another unpublished code and desire to maintain a competitive advantage to its proprietary nature or institutional review board restrictions [69]. Without the training data and code, the reproducibility of machine learning is dismal.

Given the relative novelty of SBDH in predictive analytics and the lack of standardization around data sources and outcomes assessed as well as challenges related to transparency of models in the private sector, models that incorporate SBDH factors are fraught with questions about accuracy. The lack of transparency makes it very difficult to assure model accuracy, precludes replicability, and portends clinicians’ mistrust of these models. Such challenges highlight the need for greater transparency in model development and sharing across institutions.

## Recommendations to Address Challenges and Improve SBDH Predictive Models

Advancing SBDH predictive analytics will require overcoming several challenges. As the field of health care predictive modeling grows, the incorporation of SBDH factors into predictions will face challenges similar to those of traditional models. Predictive models should follow guidelines in the *Transparent Reporting of a multivariate prediction model for Individual Prognosis or Diagnosis* (TRIPOD) initiative [71]. The TRIPOD guidelines are concerned with how general health care predictive models are reported and serve as the framework for predictive model development, validation, and modification in health care contexts [71]. This initiative was developed in response to the growing field of health-related predictive analytics and concerns about the lack of transparency, standardization, and oversight [72]. As the field of health care predictive analytics matures, it is time to apply the TRIPOD initiative’s guidelines to this rapidly evolving area of health services analytics regarding SBDH factors. Consequently, we offer several recommendations to advance the use of SBDH in health and health care predictive analytics (Textbox 1).

**Recommendations to advance the use of social and behavioral determinants of health in health care predictive analytics.**

**Privacy standards, patient consent, and ethical use of social and behavioral determinants of health (SBDH) data**

- Develop consensus on transparency, privacy protections, and ethical uses of SBDH data in predictive models
- Create guidelines to reduce inherent bias in predictive models

**Technical challenges associated with SBDH data sources and analytics**

- Determine best practice guidelines for SBDH data sources and predictive model design as well as open-source access
- Expand standardized coding and taxonomies of SBDH risk factors that enhance interoperability

**Expanding the knowledge base to inform best practice guidelines for SBDH analytics**

- Support national shared research and development to advance the SBDH predictive model development and application
- Establish a national agenda to create a shared evidence base regarding the importance of SBDH factors and the best approach for including SBDH in analytics

### Privacy Standards, Patient Consent, and Ethical Use of Social and Behavioral Determinants Data

#### Develop Consensus on Transparency, Privacy Protection, and Ethical Uses of SBDH Data in Predictive Models

As expected, many consumers are unsettled by the unregulated use of personal and commercial information to predict sensitive behaviors or health outcomes [4]. An example of such unregulated use of personal information is Google’s acquisition of large amounts of personal health data, from hospitals and clinics across 21 US states, used to predict health and health care use, undisclosed to patients and other parties [73,74]. Social determinants cover sensitive topics, such as poverty, substance misuse, food insecurity, and homelessness. Individuals may fear stigmatization from health providers in revealing their SBDH information [75]. Similarly, individuals may be concerned about the social, employment, and legal effects of revealing SBDH when their data are not protected [75].

To address such concerns, there needs to be an established discourse leading to a national consensus and clear guidelines regarding the ethical use of patients’ SBDH data in the context of a health care predictive model [76]. Lack of transparency in methods, applications, and data protection results in little accountability to ensure that SBDH risk predictions are not used to achieve profits at the expense of health care quality or access, such as using SBDH data to exclude vulnerable patients from a health intervention to ensure greater health care profits [76,77]. Establishing robust and meaningful national guidelines for using SBDH data will require insights from a variety of clinical, social science, and technical perspectives as well as views of patients, community members, policy makers, and ethicists. In particular, patients should participate and be involved in the research that is developing models to safeguard the ethical and transparent use of patient data [78]. Without the perspectives of patients and community members at the forefront of these discussions, rather than moving to a new level of health care equity and access, SBDH predictive analytics could easily slide into domains that many would consider inappropriate use, especially given a special concern and focus on the highest risk members of our communities [76].

#### Create Guidelines to Reduce Inherent Bias in Predictive Models

One important ethical and technical challenge of SBDH analytics, mostly in the application of statistical modeling, is ingrained model bias. For instance, vulnerable patients, such as those with more social and behavioral risk factors, may not be adequately represented in the data sources used to build the predictive model, leading to the model’s inaccurate predictions for these individuals. Machine learning models on the other hand can address this issue through over- or undersampling. Therefore, being at risk for bias from the original sample is normally corrected in a standard process [79].

The data sources might also lack information on the key SBDH variables that affect the desired outcomes. An example of this challenge might be a predictive model that focuses on health care utilization as the desired outcome and lacks data on health care access for vulnerable populations. Such a model may indicate that individuals with poor access to health care have a low likelihood of future utilization. A model with such ingrained bias would thus underestimate the actual requirement for the greater amount of health care resources necessary to achieve the same health outcomes once these individuals have access to health care [42]. Recently, this situation was observed in a study by Obermeyer et al [80] who assessed a large, commercial health plan’s predictive algorithm. The model systematically underestimated the health needs of African American patients by assuming that health care costs served as an adequate proxy for health needs. The bias arose because the unequal access to care among African American patients resulted in less money spent caring for those patients compared with White patients.

Although many researchers use health care utilization and costs as outcomes for SBDH research, models with these outcomes, proxied for health needs, are biased in that the data underrepresents those with lower access to health care. In recognition of the ingrained model bias, one approach might be to develop guidelines that recommend stratifying the population for key SBDH risk factors. Therefore, separate models would assess health care utilization for each stratum, taking into account unmeasured SBDH risk factors impacting health care utilization (eg, socioeconomic status, which defines insurance type and access to health care).

### Technical Challenges Associated With Data Sources and Analytics

#### Determine Best Practice Guidelines for SBDH Data Sources and Predictive Model Design As Well As Open-Source Access

The future of SBDH-centric predictive modeling faces several challenges related to data sources and model design. One *big data*–related challenge is that most social and behavioral data found within providers’ EHRs are unstructured, free-text clinical notes and are not standardly interoperable. Although ubiquitous, this information is captured inconsistently and depends on the use of NLP to render the data useful in analytics [81,82]. When NLP is utilized, the SBDH language in the health record may not describe the level of SBDH precisely enough to accurately determine social risk as social determinants such as neighborhood disadvantage may need to reach a threshold to have a significant impact on health-related outcomes [83].

Another important challenge is related to the use of population-level SBDH variables and whether such variables are interpreted as proxies for individual-level factors that cannot be measured, such as low household income, or represent population-level spatial elements, such as a high concentration of low household income in a neighborhood [84]. Proxies are based on assumptions to confer population-level characteristics to an individual. In contrast, geospatial models investigate population-level elements based on the principle of spatial autocorrelation, meaning that data located close together are interrelated by nature [85]. Addressing this challenge is critical to the interpretation of models and requires sufficiently transparent models that allow the proper distinction between the two implications of the population-level SBDH variables.

There are also several technical challenges related to the analytic approach, spanning the choice of analytic model, data sources, discriminatory power, and SBDH temporality. Statistical models, spatial analysis, and machine learning have all been used alone and in combination with various SBDH predictive models. Most often, health care predictive analytics uses regression models for their simplicity and acceptability [86]. However, machine learning models may be useful for finding new dimensions that can accurately classify outcomes according to their predictive characteristics in nonlinear data [86]. However, not all machine learning techniques, which range from transparent decision tree algorithms to unsupervised neural networks, are appropriate for use with SBDH predictive models. Highly autonomous machine learning models may select characteristics that are not clinically relevant for the outcome (eg, family meetings as a predictive characteristic for hospital mortality) when researchers do not remove these characteristics [86]. Models should instead reflect appropriate domain expertise as well as appropriate machine learning techniques. Moreover, for techniques that depend on unsupervised neural networks, there are long-standing controversies regarding the disadvantages of nontransparent, one-of-a-kind models versus more readily explainable logistic regression models [7,86].

There are also challenges related to using SBDH data at the geographic level in predictive modeling, which are often needed to identify SBDH on a population level and for community-level interventions [26]. Geospatial analysts need to choose the appropriate granularity for a model, which may be associated with a model’s discriminatory power to help distinguish those at high- versus low-risk levels [87]. Furthermore, analyzing SBDH data at different geographic levels (eg, census block group, census tract, county, and state) is methodologically complex.

The discriminatory power to distinguish patients with and without social needs also poses a challenge in nongeospatial modeling with the potential to introduce higher-than-desirable false positives and/or negatives [74]. For instance, a study of food security among Medicare patients using clinical data and a needs assessment survey could not accurately predict which patients would benefit from a referral to community resources [88]. Similarly, a predictive model that uses random forest decision methods applied to socioeconomic data did not improve referral rates to community services once at-risk patients were identified [28]. When SBDH data are operationalized in a poorly functioning algorithm, these false positives and negatives indicate that a health system spends unnecessary resources evaluating several patients not at high risk, whereas groups of patients needing social services remain unidentified [74,89]. To address this phenomenon, algorithms may need to be tested with new data as predictive analytics methods that use SBDH risk data have evidenced limited generalizability outside of the original sample data where the model was developed [26,46].

Within a model’s discriminatory power is the challenge of temporality in analytic models. Specifically, further research and development are necessary to determine how to capture changing social risk factors related to changing life circumstances throughout a person’s life or epoch [90]. For example, by structural design, a model may overlook an individual’s loss of income through unemployment or community changes not reflected in neighborhood data [74]. Thus, time-oriented models will be better able to elucidate the persistence or amelioration of disparities.

Further guidance on analytic challenges, such as optimizing the appropriate separation of high- and low-risk cases, will be crucial as part of future, wide-scale dissemination of SBDH-focused predictive modeling tools. To advance predictive analytics and increase generalizability across the United States, there should also be open-source SBDH resources for methods and databases that leverage previous SBDH research and development [91,92]. Globally, the Research Data Alliance could create a working group to spearhead the creation of open-source SBDH data sources and facilitate work toward interoperability [93].

#### Expand Standardized Coding and Taxonomies of SBDH Risk Factors That Enhance Interoperability

Once a single health care system renders SBDH data useful through advanced data science, they must find ways to disseminate these advances. The lack of standardization of SBDH data and collection processes prevents the interoperability and integration of modeling into diverse platforms [91,92] and impacts the creation of SBDH products for EHRs [94]. For greater interoperability, we need a standard, practical coding system for SBDH factors that goes beyond vendor-specific coding [91,92]. Such an endeavor is presently being pioneered by the Social Interventions Research and Evaluation Network through the HL7 *Gravity Project* [95].

### Expanding the Knowledge Base to Inform Best Practice Guidelines

#### Support National Shared Research and Development to Advance SBDH Predictive Model Development and Application

In recognition of the emerging field of SBDH predictive analytics, steps toward developing consensus and further evaluative work are needed to produce best practice guidelines for the use of SBDH data in predictive modeling [91]. There is wide variability in the choice of data sources, risk factors, targeted outcomes, geographic levels, and analytic approaches in the SBDH predictive models. Each of these model components can impact a tool’s accuracy and appropriateness for use in a particular setting or context. At present, there is a very limited understanding of the impact of these parameters on the effectiveness of the SBDH predictive model. Although endpoints such as health care cost and utilization may seem similar, the choice of health outcome in a model can obscure the path from social risk to health. Best practice guidelines should include transparency of model validation methods for various outcomes to ensure that modeling methods can be replicated in other populations [91]. The use of SBDH variables in predictive modeling is relatively new. Developing consensus might be premature in such circumstances and evaluative work must occur beforehand. However, to form guidelines, it is critical to consider standardization in SBDH predictive analytics and to organize the discourse early on. Such discourse would facilitate data sharing, create open-source tools and algorithms, and set expectations.

#### Establish a National Agenda to Create a Shared Evidence Base Regarding the Importance of SBDH Factors and the Best Approach for Including SBDH in Analytics

Although the methods and analyses addressing SBDH have matured substantially over the past decades, an expanded data infrastructure and more research are necessary to gain a full understanding of how SBDH manifests throughout a person’s life [96]. Present health analytics platforms are generally not built to advance our knowledge base in this area. Rather, they are often intended to give health systems or insurers a leg-up over their competition in achieving financial or pay-for-performance targets. There should be a national agenda to develop and share technology and human resources and strategies to support efficient data extraction, evidence-based development, and effective analytics and reporting within and across institutions in the United States [92]. For-profit entities also have a vested interest to create better predictive models. Such shared desire would be an incentive for them to participate in the development of a shared evidence base, resulting in the creation of better predictive models.

### Conclusions

In the face of great challenges and perhaps even greater benefits, we have identified a series of potential approaches for advancing the present state of predictive analytics within the SBDH context. The future of predictive modeling involving SBDH will require key stakeholders—including policy makers, payers, providers, researchers and analysts, patients, and their advocates—to reach a consensus regarding ethical frameworks, data sharing, technical parameters, and model transparency. Such a consensus will help ensure that the ultimate promise of SBDH analytics, improving health and reducing health disparities, is achieved in health care systems and communities across the United States.

## Abbreviations

CDC: Centers for Disease Control and Prevention
EHR: electronic health record
NLP: natural language processing
SBDH: social and behavioral determinants of health
TRIPOD: Transparent Reporting of a multivariate prediction model for Individual Prognosis or Diagnosis
